# Role of EP_2_ and EP_4_ receptors in airway microvascular leak induced by prostaglandin E_2_

**DOI:** 10.1111/bph.13400

**Published:** 2016-02-18

**Authors:** Victoria C Jones, Mark A Birrell, Sarah A Maher, Mark Griffiths, Megan Grace, Valerie B O'Donnell, Stephen R Clark, Maria G Belvisi

**Affiliations:** ^1^ Respiratory Pharmacology, National Heart and Lung Institute, Faculty of MedicineImperial College LondonLondonUK; ^2^ MRC‐Asthma UK Centre in Allergic Mechanisms of AsthmaLondonUK; ^3^ Leukocyte Biology, National Heart and Lung InstituteImperial College LondonLondonUK; ^4^ Institute of Infection and Immunity and Systems Immunity Research InstituteLondonUK; ^5^ Wales School of Pharmacy and Pharmaceutical SciencesCardiff UniversityCardiffUK

## Abstract

**Background and Purpose:**

Airway microvascular leak (MVL) involves the extravasation of proteins from post‐capillary venules into surrounding tissue. MVL is a cardinal sign of inflammation and an important feature of airway inflammatory diseases such as asthma. PGE_2_, a product of COX‐mediated metabolism of arachidonic acid, binds to four receptors, termed EP_1–4_. PGE_2_ has a wide variety of effects within the airway, including modulation of inflammation, sensory nerve activation and airway tone. However, the effect of PGE_2_ on airway MVL and the receptor/s that mediate this have not been described.

**Experimental Approach:**

Evans Blue dye was used as a marker of airway MVL, and selective EP receptor agonists and antagonists were used alongside EP receptor‐deficient mice to define the receptor subtype involved.

**Key Results:**

PGE_2_ induced significant airway MVL in mice and guinea pigs. A significant reduction in PGE_2_‐induced MVL was demonstrated in *Ptger2* ^*−*/*−*^ and *Ptger4* ^*−*/*−*^ mice and in wild‐type mice pretreated simultaneously with EP_2_ (PF‐04418948) and EP_4_ (ER‐819762) receptor antagonists. In a model of allergic asthma, an increase in airway levels of PGE_2_ was associated with a rise in MVL; this change was absent in *Ptger2* ^*−*/*−*^ and *Ptger4* ^*−*/*−*^ *mice.*

**Conclusions and Implications:**

PGE_2_ is a key mediator produced by the lung and has widespread effects according to the EP receptor activated. Airway MVL represents a response to injury and under ‘disease’ conditions is a prominent feature of airway inflammation. The data presented highlight a key role for EP_2_ and EP_4_ receptors in MVL induced by PGE_2._

**Abbreviations**
BAFL: bronchiolar lavage fluid
COPD: chronic obstructive pulmonary disease
IPA: intrapulmonary airways
MVL: microvascular leakage
OVA: ovalbumin

## Tables of Links

**Table:** 

**TARGETS**
EP_1_ receptor
EP_2_ receptor
EP_3_ receptor
EP_4_ receptor

**Table:** 

LIGANDS	
5‐HT	ONO‐AE1‐259
Diclofenac	ONO‐AE1‐329
ER‐819762	PGE_2_
Evans blue dye	PF‐04418948
ONO‐AE‐248	

These Tables list key protein targets and ligands in this article which are hyperlinked to corresponding entries in http://www.guidetopharmacology.org, the common portal for data from the IUPHAR/BPS Guide to PHARMACOLOGY (Pawson *et al.*, 2014) and are permanently archived in the Concise Guide to PHARMACOLOGY 2015/16 (Alexander *et al.*, 2015).

## Introduction

Airway microvascular leakage (MVL) and plasma exudation represent classical features in the pathogenesis of various respiratory diseases, including asthma and chronic obstructive pulmonary disease (COPD) (Paredi and Barnes, 2009). The bronchial microvasculature has a multitude of important functions that are essential for maintaining pulmonary homeostasis, but during an inflammatory response this barrier can be disrupted allowing fluid and large macromolecules to move into the surrounding tissues through interendothelial gaps. Many mediators (e.g. cysteinyl leukotrienes, histamine, bradykinin, 5‐HT and cytokines) are capable of inducing this effect when released in response to an inflammatory insult in the airway, where they can act upon the endothelium of post‐capillary venules to open these intercellular gaps. This effect causes plasma to ‘leak’ out into extravascular sites because of hydrostatic pressure gradients (Olivenstein *et al.*, 1997; Reynolds *et al.*, 2002; Greiff *et al.*, 2003). This phenomenon is a very distinctive feature of acute inflammation but is also observed in more chronic diseases such as asthma (Laitinen *et al.*, 1987; Li and Wilson, 1997; Innes *et al.*, 2009; Khor *et al.*, 2009) and COPD (Hill *et al.*, 1999; Minakata *et al.*, 2005; Bessa *et al.*, 2012).

PGE_2_ is an endogenous lipid eicosanoid synthesized by COX‐mediated metabolism of free arachidonic acid. It is produced in a variety of cells, including airway smooth muscle, epithelial cells, alveolar macrophages and pulmonary endothelial cells (Meyrick *et al.*, 1989; Widdicombe *et al.*, 1989). It exerts its biological effects via activation of four cell‐surface GPCRs, termed EP_1–4_, encoded for by the genes *Ptger1–4* (Coleman *et al.*, 1994). Increased levels of PGE_2_ have been reported in bronchiolar lavage fluid (BALF) and plasma of asthma patients (Brightling *et al.*, 2000; Birring *et al.*, 2003; Long *et al.*, 2004; Sastre *et al.*, 2008), allergen‐challenged mice (Herrerias *et al.*, 2009) and enhanced levels in the exhaled breath condensate of COPD patients (Montuschi *et al.*, 2003; Chen *et al.*, 2008; Antczak *et al.*, 2012).

Previous work from our lab, and others, has demonstrated that the bronchodilator effects of PGE_2_ occur through the activation of the EP_4_ receptor (Buckley *et al.*, 2011; Benyahia *et al.*, 2012), whereas airway sensory nerve activation and cough appear to be via the activation of the EP_3_ receptor (Maher *et al.*, 2009). However, the effect of PGE_2_ and the EP receptors on airway MVL has not been extensively investigated. To investigate this, Evans Blue dye was used as a marker of MVL, a method that has been used for decades to quantify vascular permeability in various tissues and a variety of species (Miles and Miles, 1952; Evans *et al.*, 1987; Rogers *et al.*, 1988; Baluk *et al.*, 1999; Reynolds *et al.*, 2002; Xie *et al.*, 2003; Zhuang *et al.*, 2011). First, we established the responses to PGE_2_ in both mouse and guinea pig airways. Then using a pharmacological approach and EP receptor‐deficient mice, we provided substantial evidence that both the EP_2_ and EP_4_ receptors mediate PGE_2_‐induced airway MVL.

## Methods

### Animals

Male C57BL/6 mice (20–25 g) and male Dunkin Hartley guinea pigs (250–350 g) were purchased from Harlan (Bicester, Oxon, UK). Homozygous breeding pairs of mice genetically modified to disrupt one of the following genes: *Ptger1* (EP_1_), *Ptger2* (EP_2_) and *Ptger3* (EP_3_) (Ushikubi *et al.*, 1998), had been backcrossed at least eight times onto the C57BL/6 background. *Ptger4* ^*−*/*−*^ (EP_4_) mice do not survive on the C57BL/6 background because of patent ductus arteriosus (Segi *et al.*, 1998), so they were backcrossed on a mixed background of 129/Ola X C57bl/6. Mice were kindly provided by Dr Shuh Narumiya, Kyoto University, and breeding colonies maintained at Imperial College, London. All animals were housed in individually ventilated cages and provided with food and water *ad libitum* in a controlled environment. A 12 h light–dark cycle was maintained for all animals. All studies and procedures were approved by the Imperial College, Animal Welfare and Ethical Review Body, and performed in accordance with Home Office guidelines under the Animals (Scientific Procedures) Act of 1986 and the ARRIVE guidelines (Kilkenny *et al.*, 2010) and the editorial on reporting animal studies (McGrath and Lilley, 2015).

### General methodology for measuring microvascular leak

Apart for the data generated in the various mouse knockout line, animals were randomised as regards the treatment groups. Animals were anaesthetized with i.p. urethane (2 g·kg^−1^; 200 μL of a 25% solution given at two sites, followed by a further 50–100 μL as required). Mice breathe spontaneously under urethane and so were therefore not artificially ventilated. Depth of anaesthesia was assessed using the pedal and corneal reflex. Once surgical anaesthesia was reached, the jugular veins were then exposed following a midline incision over the thorax. The administration of substances i.v. was achieved by passing the injection needle through the pectoralis major as this helped prevent bleeding on withdrawal. Animals received Evans Blue dye (20 mg·kg^−1^) followed by the specific mediator or its vehicle 1 min later (4 mL·kg^−1^). The doses and timings used were those shown to be effective in previous studies within the group and from the literature (Hele *et al.*, 2001; Belvisi *et al.*, 2009). Thirty minutes after vehicle or compound administration, the animals were killed by an overdose of anaesthesthetic (pentobarbitone 200 mg kg^−1^, i.p.), the thoracic cavity was opened and a small incision was made in the left ventricle and also the left atrium of the heart. A cannula was then inserted into the left ventricle and the systemic circulation perfused with sterile saline (0.9%) at a pressure of approximately 100 mmHg. This was continued until the perfusate ran clear (2–5 min). The purpose of this was to remove any intravascular dye. The heart, lungs and oesophagus were removed *en bloc* and then the trachea and lungs separated from the heart and oesophagus. The oesophagus and bladder were taken for the initial studies and used as non‐airway, reference tissues. The trachea was isolated by cutting just above the bifurcation of the bronchi and the larynx removed. The parenchyma was then carefully scraped off using a scalpel to reveal the intrapulmonary airways (IPA). The trachea, the bronchi and IPA, the oesophagus and the bladder were then all weighed, and the wet tissue weight was recorded. Each tissue was then incubated in 120 μL of formamide at 37.5°C for at least 18 h to facilitate the extraction of Evans Blue dye. The concentration of Evans Blue extracted from each tissue was determined by light absorbance at 620 nm using a spectrophotomer; 100 μL of formamide was removed from each Eppendorf and pipetted into a 96‐well plate alongside a standard curve of Evans Blue in formamide (0, 0.3125, 0.625, 1.25, 2.5, 5, 10 and 20 μg·mL^−1^). The concentration was then calculated by interpolation from the standard curve and expressed as ng·mg^−1^ of tissue. End points were assessed by a different operator than the experimental part of the study.

### Experimental design

#### PGE_2_‐induced airway microvascular leak

A dose–response curve to PGE_2_ was established where male C57BL/6 mice were given PGE_2_ (0.1, 0.3, 1, 3 or 10 mg·kg^−1^ at 4 mL·kg^−1^) and 5‐HT (10 mg·kg^−1^ at 4 mL·kg^−1^) as a positive control or vehicle (1% ethanol in saline). Thirty minutes after administration, Evans Blue extravasation was measured. A non‐selective COX inhibitor, diclofenac (30 mg·kg^−1^ in 10 mL·kg^−1^) (Mitchell *et al.*, 1993), or vehicle (0.5% methylcellulose, 0.2% Tween 80 in water) was administered orally 60 min prior to PGE_2_ administration to block endogenous prostanoids that may influence the baseline or PGE_2_‐induced response. Having assessed the effect of i.v. PGE_2_, administration via topical delivery was then investigated to mimic what might happen in airway disease. Here, mice were anaesthetized and then dosed intra‐nasally with either vehicle (1% ethanol in saline) or PGE_2_ (3 mg·kg^−1^) in 50 μL. Once surgical anaesthesia was reached, mice were then given Evans Blue and killed 30 min later.

Guinea pigs were chosen as a second (larger) species to assess PGE_2_‐induced airway MVL as they more closely resemble humans in terms of their respiratory anatomy (lobing and branching) and physiology (mediator release). Male Dunkin–Hartley guinea pigs (300–600 g) were anaesthetized with urethane (2 g·kg^−1^ i.p. of a 25% solution). Once surgical anaesthesia was reached, animals received Evans Blue (20 mg·mL^−1^ at 1 mL·kg^−1^ i.v.) and then 1 min later vehicle (1% ethanol in saline), PGE_2_ (3 mg·kg^−1^) or 5‐HT (1 mg·kg^−1^), all i.v. at 1 mL kg^−1^; 5 min after 5‐HT or 30 min after PGE_2_, the extravasation of Evans Blue was measured.

#### Identifying the EP receptor mediating PGE_2_‐induced airway microvascular leak: EP receptor‐deficient mice

The same MVL protocol was used as before with a single submaximal dose of PGE_2_ (3 mg·kg^−1^) or vehicle (1% ethanol in saline) used. Additionally, the response to 5‐HT in EP receptor knockouts (*ptger2* ^*−*/*−*^ and *ptger4* ^*−*/*−*^) and their wild types was investigated to ensure that their lack of response to PGE_2_ was not due to an overall disruption in MVL. A submaximal dose of 5‐HT (1 mg·kg^−1^) was used as a known inducer of MVL.

#### Identifying the EP receptor mediating PGE_2_‐induced airway microvascular leak: selective EP receptor agonists

To parallel the effects seen in the EP receptor‐deficient mice, the response to selective EP agonists was studied. Following Evans Blue administration, animals were given selective EP receptor agonists (Okada *et al.*, 2000; Suzawa *et al.*, 2000; Cao *et al.*, 2002): ONO‐D1‐004 (EP_1_), ONO‐AE1‐259 (EP_2_), ONO‐AE‐248 (EP_3_), ONO‐AE1‐329 (EP_4_) or PGE_2_ (3 mg·kg^−1^) i.v. at 4 mL·kg^−1^ or vehicle (1% ethanol in saline).

#### Inhibition of EP_2_‐ and EP_4_‐induced microvascular leak using selective EP_2_ and EP_4_ receptor antagonists

Selective receptor antagonists were used to confirm data generated with selective agonists. Initially, a dose response to ONO‐AE1‐259 (EP_2_ agonist) was performed to establish a suitable dose to use. The doses used were 0.1, 0.3, 1 and 3 mg·kg^−1^ (i.v. at 4 mL·kg^−1^). The EP_2_ antagonist, PF‐04418948, was used in subsequent studies and was dosed at 3, 10, 30 or 100 mg·kg^−1^ (10 mL·kg^−1^ i.p.). A vehicle control was also included (0.5% methylcellulose, 0.2% Tween 80 in saline). Mice were dosed with PF‐04418948 (af Forselles *et al.*, 2011) or vehicle 1 h before ONO‐AE1‐259 administration (3 mg·kg^−1^ in 4 mL·kg^−1^ i.v.). Next, a dose–response curve to ONO‐AE1‐329 (EP_4_ agonist) was carried out using 0.1, 0.3, 1 and 3 mg·kg^−1^ (i.v. at 4 mL·kg^−1^). The EP_4_ antagonist, ER‐819762 (Chen *et al.*, 2010), was used in subsequent studies and was dosed at 3, 10, 30 or 100 mg·kg^−1^ (10 mL·kg^−1^ i.p.). A vehicle control was also included (0.5% methylcellulose, 0.2% Tween 80 in saline). Mice were dosed with ER‐819762 or vehicle 1 h before ONO‐AE1‐329 administration (3 mg·kg^−1^ in 4 mL·kg^−1^ i.v.).

#### Inhibition of PGE_2_‐induced microvascular leak by EP_2_ and EP_4_ receptor antagonists

Mice were dosed with PF‐04418948 (10 mg·kg^−1^ at 10 mL·kg^−1^ i.p.) or ER‐819762 (30 mg·kg^−1^ at 10 mL·kg^−1^ i.p.) or vehicle (0.5% methylcellulose, 0.2% Tween 80 in saline) 1 h before PGE_2_ administration (3 mg·kg^−1^ at 4 mL·kg^−1^ i.v.). Additionally, in a separate study, mice were dosed with both PF‐04418948 and ER‐819762 simultaneously 1 h before PGE_2_.

#### Identifying the EP receptors involved in ovalbumin‐induced microvascular leak: EP receptor‐deficient mice

Mice were sensitized with ovalbumin (OVA) (10 μg per mouse, 100 μL i.p.) on days 0 and 14 and on days 24, 25 and 26 mice were intranasally challenged with saline or OVA (50 μg in 50 μL) under isoflurane anaesthesia. PGE_2_ levels in the BALF were measured using LC‐MS/MS following lipid extraction as follows: 5 ng PGE_2_‐d4 was added to samples before extraction, as an internal standard. Lipids were extracted by adding a solvent mixture (1 mol·L^−1^ acetic acid, isopropyl alcohol, hexane (2:20:30, v v^−1^v^−1^)) to the sample at a ratio of 2.5 to 1 mL, vortexing and then adding 2.5 mL of hexane. After vortexing and centrifugation, lipids were recovered in the upper hexane layer. The samples were then re‐extracted by addition of an equal volume of hexane. The combined hexane layers were dried and analysed for free or esterified PGs using LC‐MS/MS. Lipids were separated on a C18 Spherisorb ODS2, 5 μm, 150 × 4.6 mm column (Waters, Hertfordshire, UK) using a gradient of 50–90% B over 10 min (A, water : acetonitrile : acetic acid, 75:25:0.1; B, methanol : acetonitrile : acetic acid, 60:40:0.1) with a flow rate of 1 mL·min^−1^. Products were quantified by LC‐MS/MS electrospray ionization on a Sciex 4000 Q‐Trap using parent‐to‐daughter transitions of *m*/*z* 351.2 [M − H]^−^ to *m*/*z* 271 for PGE_2_ and *m*/*z* 355.2 to 275.3 for PGE_2_‐d4 with declustering potential of −55 and collision energy of −26 V. Products were identified and quantified using standards run in parallel under the same conditions.

Based on these data, MVL was measured at 2 and 24 h after the final intranasal challenge. In a subsequent study, allergy‐induced MVL was compared in wild‐type and EP receptor knockout (*ptger2* ^*−*/*−*^ and *ptger4* ^*−*/*−*^) mice.

### Compounds and materials

The EP_2_ receptor antagonist, PF‐04418948, was a gift from Nick Pullen, Pfizer (Kent, UK). The EP_4_ antagonist, ER‐819762, was a gift from Eisai (Hertfordshire, UK). The EP_1_ receptor agonist (ONO‐D1‐004), the EP_2_ receptor agonist (ONO‐AE1‐259), the EP_3_ receptor agonist (ONO‐AE‐248) and the EP_4_ receptor agonist (ONO‐AE1‐329) were gifts from Ono Pharmaceuticals (Osaka, Japan). PGE_2_ was purchased from Cayman Europe (Tallinn, Estonia). Both antagonists were made up in 0.5% methylcellulose, 0.2% Tween 80 in saline (or water for p.o. administration). PGE_2_ and all agonists were made up in 1% ethanol in saline. All other chemicals and reagents were from Sigma Aldrich (Poole, UK).

### Data analysis and statistical procedures

All data were analysed using GraphPad Prism 5. Data are expressed as mean ± SEM. Multiple measurements were analysed by one‐way ANOVA; non‐parametric Kruskal–Wallis test and *post‐hoc* comparisons were performed by Dunn's multiple comparison test, comparing selected columns to a control. Additionally, an unpaired *t*‐test and/or non‐parametric Mann–Whitney test, with two‐tailed *P* values, was carried out, where appropriate, to determine statistical significance between two groups. Differences were considered statistically significant if *P* < 0.05. The figures have been graphically presented on a range‐specific axis. The data and statistical analysis comply with British Journal of Pharmacology guidelines (Curtis *et al.*, 2015).

## Results

### PGE_2_‐induced airway microvascular leak

**Figure 1:**
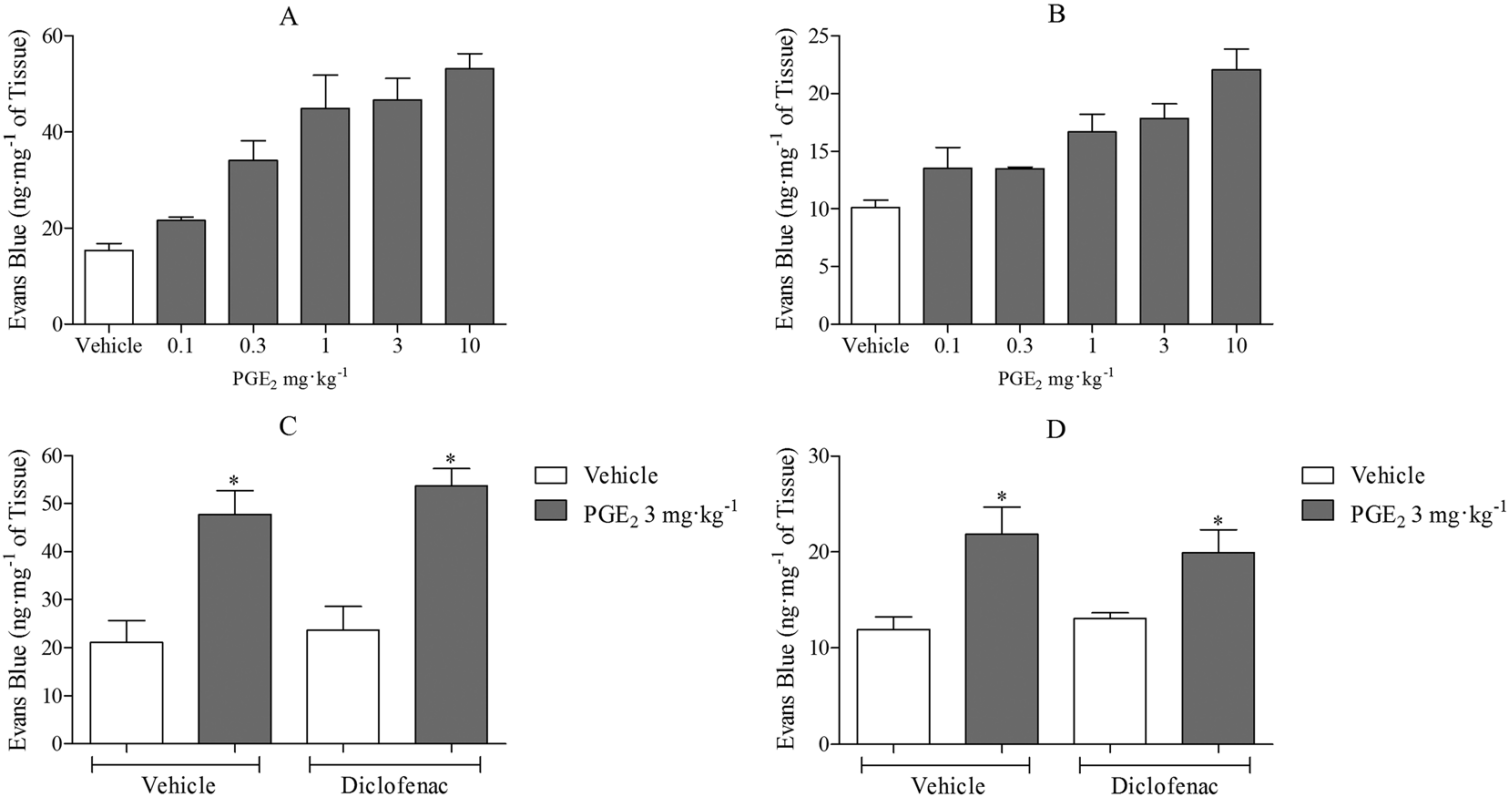
The effect of PGE_2_ on MVL in the trachea and IPA of C57BL/6 mice. (A) Dose–response to PGE_2_ (0.1–10 mg·kg^−1^ i.v., *n* = 3–4) after 30 min (A: trachea, B: bronchi and IPA). Effect of diclofenac (30 mg·kg^−1^ in 10 mL·kg^−1^ p.o.; 1 h) on MVL in vehicle and PGE_2_ (3 mg·kg^−1^ i.v.; 30 min) treated mice (*n* = 4) (C: trachea, D: bronchi and IPA). Data expressed as mean ± SEM of the concentration of Evans Blue dye (ng·mg^−1^ of tissue). *P < 0.05 indicates significance of treatment groups from vehicle control.

**Figure 2:**
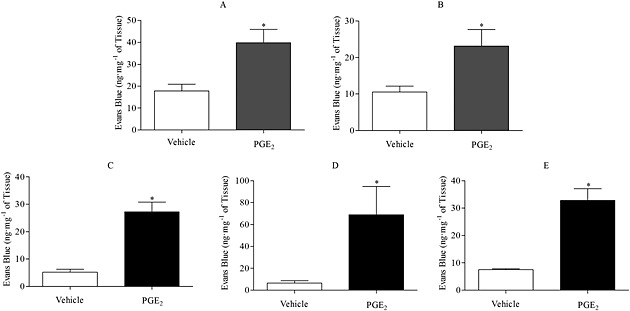
Effect of an intranasal dose of PGE_2_ (3 mg·kg^−1^ intranasally, *n* = 6) on MVL after 30 min into the trachea (A) or bronchi and IPA (B) of mice. Effect of PGE_2_ (3 mg·kg^−1^ i.v.) on MVL into the upper trachea (C), lower trachea (D) or bronchi and IPA (E) of Dunkin–Hartley guinea pigs. Data expressed as mean ± SEM of the concentration of Evans Blue dye (ng·mg^−1^ of tissue). *P < 0.05 indicates significance of treatment groups from vehicle control.

PGE_2_ (0.1, 0.3, 1, 3, 10 mg·kg^−1^, i.v.) induced dose‐dependent MVL in both the trachea and the bronchi and IPA (Fig. 1A, B). From this, a submaximal dose of 3 mg·kg^−1^ was chosen. No statistically significant increase in MVL was detected in either the bladder or oesophagus after PGE_2_ challenge [vehicle = 58.1 ± 20.3 vs. PGE_2_ = 46.5 ± 21.2, vehicle = 25.4 ± 15.1 vs. PGE_2_ = 32.2 ± 8, Evans Blue (ng·mg^−1^ of tissue), respectively]. Diclofenac, a nonselective COX inhibitor, was administered prior to PGE_2_ administration to block the production and release of endogenous prostanoids to ensure that they were not influencing baseline or PGE_2_‐induced airway leak. The data indicate that diclofenac had no effect on baseline or PGE_2_‐induced leak in the trachea or bronchi and IPA (Fig. 1C, D). In both the vehicle‐ and diclofenac‐treated groups, PGE_2_ significantly increased airway MVL. Having established that i.v. PGE_2_ induced MVL in murine airways, we wanted to examine whether such a response could be replicated via topical administration direct to the airways. Therefore, a single intranasal dose of PGE_2_ (3 mg·kg^−1^) was administered and EB leak measured after 30 min. Topical PGE_2_ induced a similar MVL response to that seen with i.v administration (Fig. 2A, B). Last, to determine whether the effect seen with PGE_2_ was exclusive to the mouse, a submaximal dose of PGE_2_ was administered to guinea pigs alongside 5‐HT, acting as a positive control. PGE_2_ (3 mg·kg^−1^ i.v.) induced significant MVL in the trachea, bronchi and IPAs was comparable with 5‐HT (Fig. 2C–E).

### PGE_2_‐induced airway microvascular leak in EP receptor‐deficient mice: role for EP_2_ and EP_4_ receptors

**Figure 3:**
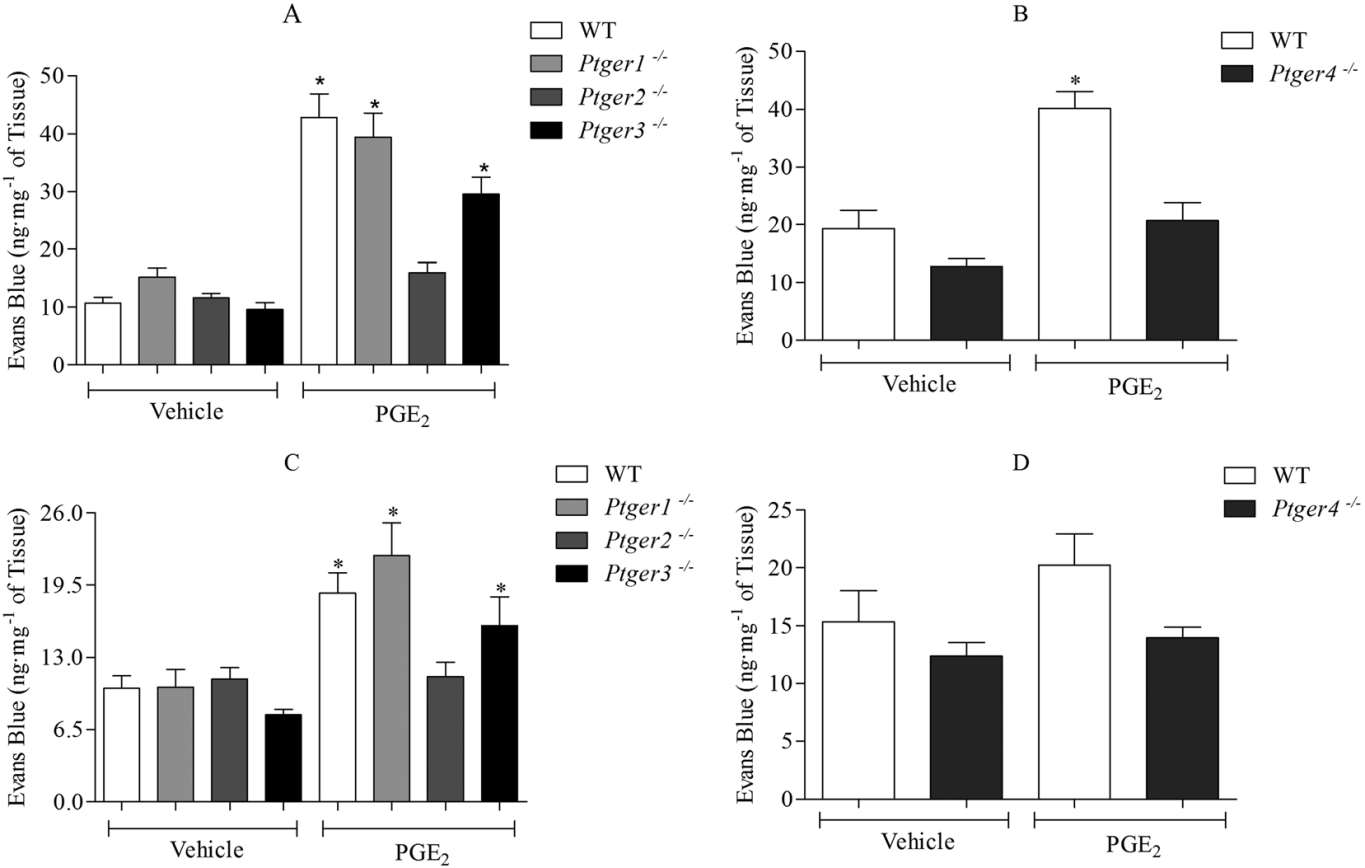
Investigating the responses to PGE_2_ (3 mg·kg^−1^ i.v., *n* = 6) in wild‐type (WT) and EP_1–4_ receptor‐deficient mice (A and B) trachea and (C and D) bronchi and IPAs. EP_1–3_ receptor‐deficient mice are bred on a C57BL/6 background, and EP_4_ receptor‐deficient mice are bred on a mixed background of 129/Ola X C57BL/6. Data expressed as mean ± SEM of the concentration of Evans Blue dye (ng·mg^−1^ of tissue). *P < 0.05 indicates significance from vehicle control.

**Figure 4:**
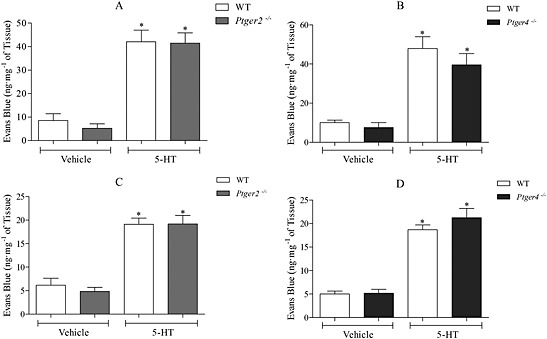
Effect of 5‐HT (1 mg·kg^−1^ i.v., *n* = 6) on MVL in wild‐type (WT) and EP_2_ or EP_4_ receptor‐deficient mice (A and B) trachea and (C and D) bronchi and IPA. EP_2_ receptor‐deficient mice were bred on a C57BL/6 background, and EP_4_ receptor‐deficient mice were bred on a mixed background of 129/Ola X C57BL/6. Data expressed as mean ± SEM of the concentration of Evans blue dye (ng·mg^−1^ of tissue). *P < 0.05 indicates a significant increase from vehicle control.

The effect of PGE_2_ (3 mg·kg^−1^) on MVL was compared in wild type and mice deficient in individual EP receptors (*Ptger1–4* ^*−*/*−*^). The data demonstrated a significant increase in MVL in wild‐type, *Ptger1* ^*−*/*−*^ and *Ptger3* ^*−*/*−*^ mice in response to PGE_2_ (Fig. 3A and C). However, a substantial and significant reduction in PGE_2_‐induced MVL was shown in *Ptger2* ^*−*/*−*^ and *Ptger4* ^*−*/*−*^ mice (Fig. 3A–D). This was apparent in the trachea and the bronchi and IPA. To determine whether their lack of response to PGE_2_ was not due to an overall disruption in MVL, 5‐HT was administered to *Ptger2* ^*−*/*−*^ and *Ptger4* ^*−*/*−*^ mice. Here, 5‐HT produced a significant increase in MVL in the trachea and in the bronchi and IPA, with no difference seen between the EP receptor‐deficient mice and the wild types (Fig. 4).

### Selective EP_2_ and EP_4_ receptor agonists induce airway microvascular leak

**Figure 5:**
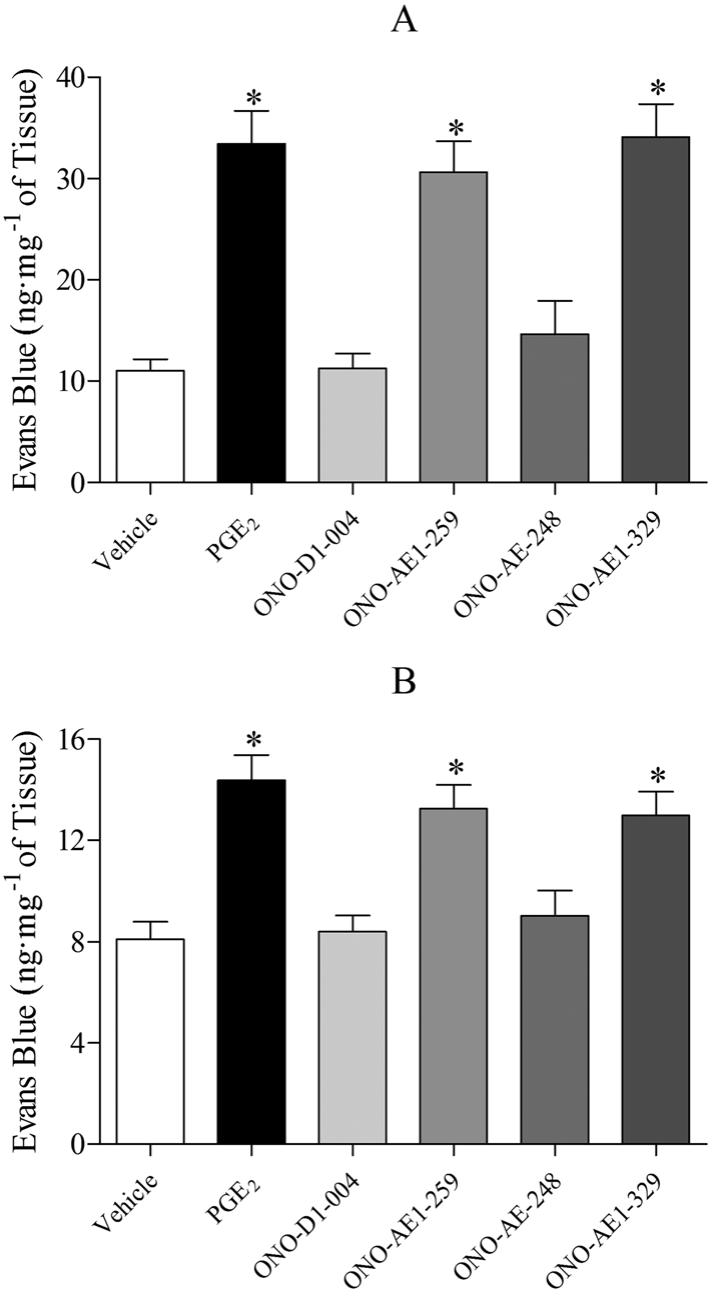
Effect of PGE_2_, ONO‐D1‐004 (EP_1_), ONO‐AE1‐259 (EP_2_), ONO‐AE‐248 (EP_3_) and ONO‐AE1‐329 (EP_4_) (3 mg·kg^−1^ i.v.; 30 min) on MVL into the trachea (A) and bronchi and IPA (B) of C57BL/6 mice. Data expressed as mean ± SEM of the concentration of Evans Blue dye (ng·mg^−1^ of tissue), *n* = 6. *P < 0.05 indicates a significant increase from vehicle control.

Having shown an inhibition in PGE_2_‐induced MVL in EP_2_ and EP_4_ receptor‐deficient mice, selective EP receptor agonists were investigated. ONO‐AE1‐259 (EP_2_) and ONO‐AE1‐329 (EP_4_) both significantly increased MVL in both the trachea and bronchi and IPA of mice, while ONO‐D1‐004 (EP_1_) and ONO‐AE‐248 (EP_3_) had little or no effect on MVL in either trachea or bronchi and IPA (Fig. 5).

### Inhibition of EP_2_‐ and EP_4_‐induced microvascular leak using selective EP_2_ and EP_4_ receptor antagonists

**Figure 6:**
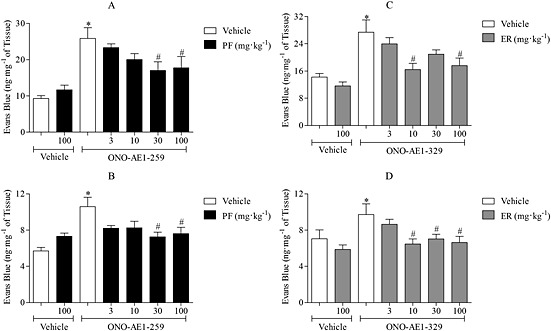
Inhibition of EP_2_‐ and EP_4_‐induced MVL using selective EP receptor antagonists. Effect of the EP_2_ receptor antagonist, PF‐04418948 (PF; 3, 10, 30 and 100 mg·kg^−1^ i.p.; 1 h), on ONO‐AE1‐259‐induced MVL (3 mg·kg^−1^ i.v.) in trachea (A) and bronchi/IPA (B). The effect of the EP_4_ receptor antagonist, ER‐819762 (3, 10, 30 and 100 mg·kg^−1^ i.p.; 1 h), on ONO‐AE1‐329‐induced MVL (3 mg·kg^−1^ i.v.) in trachea (C) and bronchi and IPA (D). Data expressed as mean ± SEM of the concentration of Evans Blue dye (ng·mg^−1^ of tissue), *n* = 6. *P < 0.05 indicates a significant increase from vehicle control. #*P* < 0.05 indicates a significant decrease from treatment control.

The EP_2_ agonist (ONO‐AE1‐259) produced a dose‐dependent increase in MVL. Similar to previous studies 3 mg·kg^−1^ induced a significant increase in MVL in the trachea (data not shown). This dose was therefore used in subsequent antagonist studies. The EP_2_ receptor antagonist, PF‐04418948 (i.p.), evoked a dose‐dependent inhibition with a dose of 30 mg·kg^−1^ showing a significant inhibition of EP_2_‐induced MVL (Fig. 6A, B). ONO‐AE1‐329 (EP_4_ agonist) produced a dose‐dependent increase in MVL with a significant increase in airway MVL at 3 mg·kg^−1^ (data not shown). The EP_4_ receptor antagonist, ER‐819762 (i.p.), was able to significantly inhibit MVL induced by ONO‐AE1‐329 (Fig. 6C, D). PF‐04418948 (30 mg·kg^−1^ i.p.) and ER‐819762 (10 mg·kg^−1^ i.p.) were selected for further study.

### Inhibition of PGE_2_‐induced microvascular leak by EP_2_ and EP_4_ receptor antagonists

**Figure 7:**
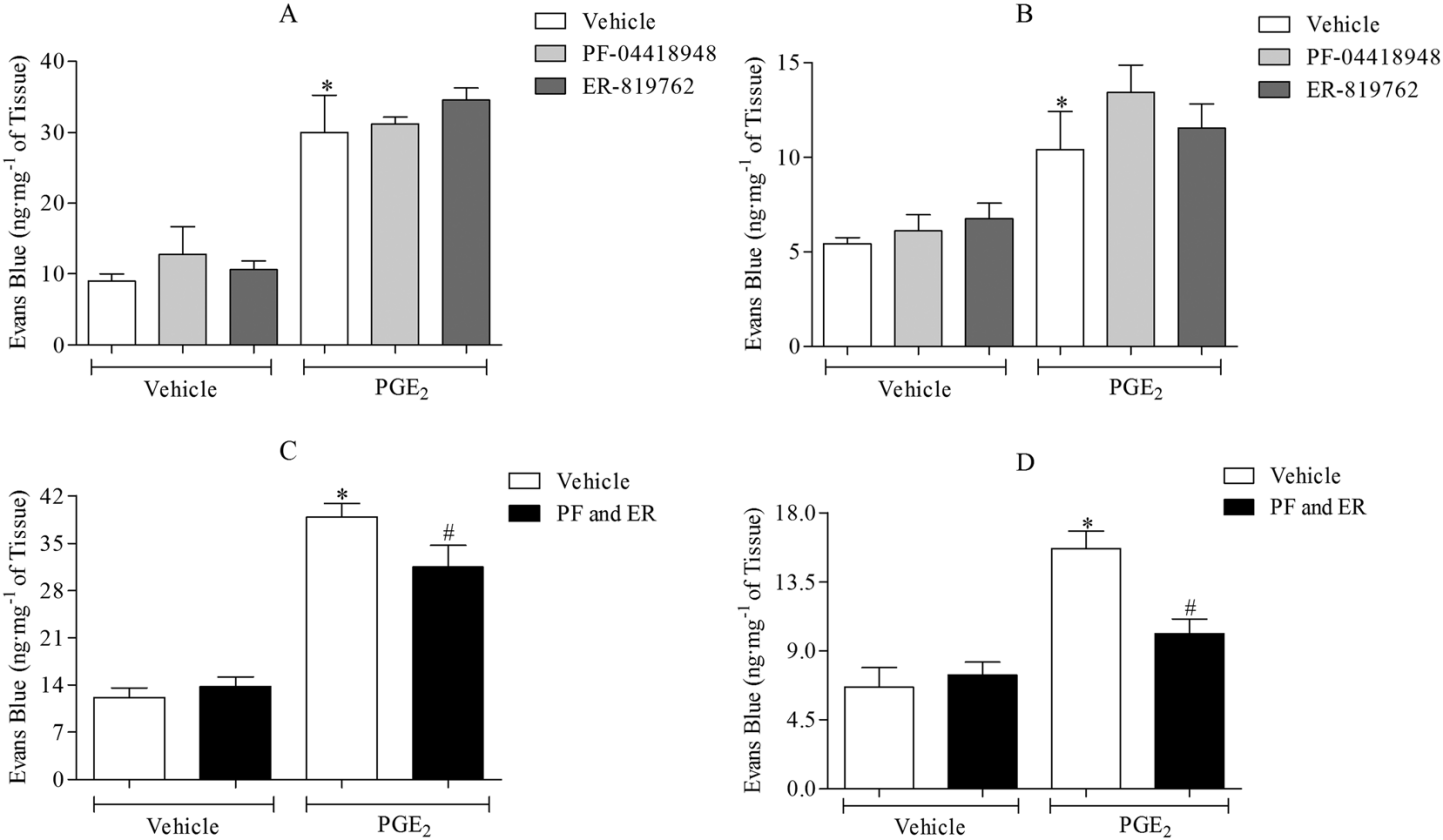
Effect of PF‐04418948 (PF; 30 mg·kg^−1^ i.p.; 1 h) and ER‐819762 (ER; 10 mg·kg^−1^ i.p.; 1 h) on PGE_2_‐induced MVL (3 mg·kg^−1^ i.v.; 30 min) in the trachea (A) and bronchi and IPA (B) of mice. In the top two graphs, each antagonist was dosed separately (*n* = 5–6), while in the bottom two graphs, both antagonists were given simultaneously to the same animal in trachea (C) and bronchi and IPA (D). Data expressed as mean ± SEM of the concentration of Evans Blue dye (ng·mg^−1^ of tissue). *P < 0.05 indicates a significant increase from vehicle control. #*P* < 0.05 indicates a significant decrease from treatment control.

The effect of PF‐04418948 and ER‐819762 on PGE_2_‐induced MVL in mouse airways was investigated. Initially, each antagonist was dosed separately; however, as demonstrated in Fig. 7A and B, neither antagonist alone inhibited PGE_2_‐induced airway MVL. When both antagonists were given simultaneously, there was a significant reduction in PGE_2_‐induced MVL in the trachea and in the bronchi and IPA (Fig. 7C, D).

### The role of PGE_2_ and EP_2_ and EP_4_ receptors in asthma‐related airway microvascular leak

**Figure 8:**
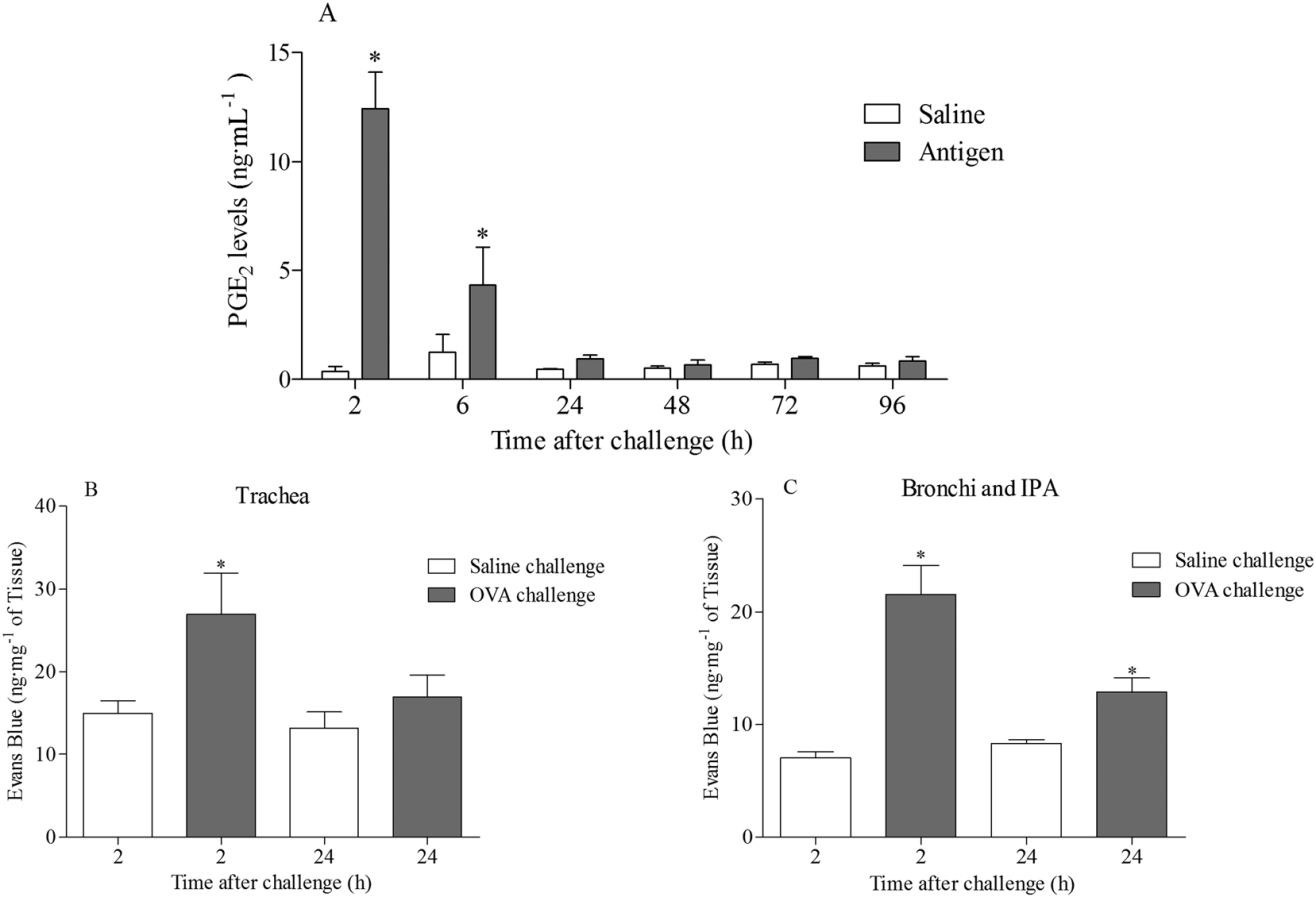
Effect of OVA challenge on BALF PGE_2_ levels (A) and microvascular leak into the trachea (B) and bronchi and IPA (C) of sensitized mice. Levels of BALF PGE_2_ were determined using LC‐MS/MS and expressed as mean ± SEM (*n* = 6). The airway MVL data are expressed as mean ± SEM of the concentration of Evans Blue dye (ng·mg^−1^ of tissue, *n* = 4). *P < 0.05 indicates a significant increase from vehicle control.

The effect of antigen challenge on BALF PGE_2_ levels in a model of allergic asthma was investigated. The antigen caused an increase in BALF PGE_2_ levels, with levels increased at 2 and 6 h after challenge and returning to basal by 24 h (Fig. 8A). This temporal increase coincided with a significant increase in airway MVL 2 h after challenge, but not after 24 h (Fig. 8B, C).

**Figure 9:**
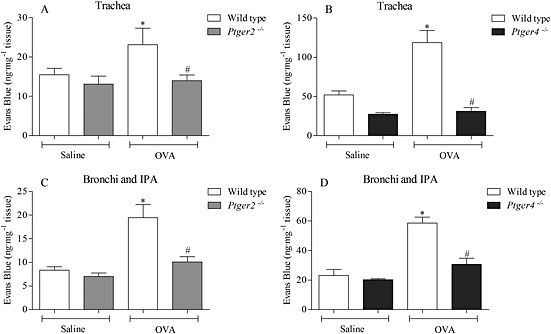
Investigating the responses to OVA challenge in sensitized, wild‐type and EP_2_ or EP_4_ receptor‐deficient mice in the (A and B) trachea and the (C and D) bronchi and IPA. Airway MVL was measured 2 h following the final intranasal antigen challenge. Airway tissue was dried before the extraction of Evans Blue dye. Data expressed as mean ± SEM of the concentration of Evans Blue dye (ng·mg^−1^ of dry tissue), *n* = 4–8. *P < 0.05 indicates a significant increase from vehicle control. #*P* < 0.05 indicates a significant decrease from wild‐type control.

The data from a follow‐up study showed that the MVL in the asthma model was absent in mice missing functional EP_2_ or EP_4_ receptors, mirroring the observations made in the exogenous PGE_2_ studies (Fig. 9A–D).

## Discussion

Airway microvascular leak is a cardinal sign of inflammation and is a prominent feature of both asthma and COPD. Various inflammatory mediators are capable of inducing this effect following an inflammatory insult to the airways. These inflammatory mediators act upon the endothelium of post‐capillary venules to open intercellular gap junctions. PGE_2_ is a ubiquitous eicosanoid that has been implicated in the pathogenesis of both asthma and COPD. Increased levels of PGE_2_ have been reported in BALF and plasma of asthma patients and the levels are enhanced in the breath condensate of COPD patients (Montuschi *et al.*, 2003; Long *et al.*, 2004). The purpose of this investigation was to establish the role of PGE_2_ in mediating airway microvascular leak and the EP receptor(s) responsible. This is an important step in understanding the biological profile of EP receptors given that PGE_2_ is a key mediator produced by the lung in health and in patients with lung disease.

Initial experiments were focused on investigating the effect of exogenous PGE_2_ in the airways of mice. PGE_2_ caused a dose‐dependent increase in MVL in the trachea and bronchi and IPA of mice, and in addition, blocking the production of endogenous prostanoids with the COX inhibitor diclofenac did not impact on PGE_2_‐induced MVL. We also observed a measurable and significant increase in MVL following an intranasal dose of PGE_2_, indicating that the effects we are seeing are not specific to the route of administration. Furthermore, we did not detect any MVL in non‐airway, reference tissues, oesophagus and bladder, suggesting that in our model system, i.v. administration of PGE_2_ mainly impacts on the airways. As the majority of research into airway MVL has been carried out in larger species, including the rat and the guinea pig, we wanted to rule out that what was being observed with PGE_2_ was not exclusive to the mouse. In the guinea pig, PGE_2_ produced a large, substantial increase in airway MVL that was comparable to that seen with 5‐HT. Both the mouse and the guinea pig produced similar profiles in the magnitude of their responses, demonstrating that the effect of PGE_2_ is translatable between species.

Having shown that PGE_2_ induced MVL in the mouse, we wanted to determine which of the EP receptors were central to this response. Differing effects of PGE_2_ have been demonstrated *in vivo*, and these, often opposing, effects can be attributed to the activation of different receptor subtypes (EP_1–4_) with different signalling characteristics (Breyer *et al.*, 2001). Using mice deficient in specific prostanoid receptors, we were able to identify the EP_2_ and EP_4_ receptor/s as mediating the inflammatory actions of PGE_2_. 5‐HT evoked MVL in wild‐type, EP_2_ and EP_4_ knockout mice, indicating that MVL was not modulated *per se* in the genetically modified mice. In addition to data generated in gene‐deleted mice, pharmacological characterisation was also performed. Experiments were performed with the four agonists, ONO‐D1‐004 (EP_1_), ONO‐AE1‐259 (EP_2_), ONO‐AE‐248 (EP_3_) and ONO‐AE1‐329 (EP_4_), and the data clearly demonstrated that, similar to PGE_2_, the EP_2_ and EP_4_ agonists significantly increase airway MVL. This confirmed that activation of these EP receptors mediated MVL in mouse airways, while activation of EP_1_ and EP_3_ receptors produced no significant increase. Interestingly, others, using an *in vitro* system with human microvascular endothelial cells, have reported that PGE_2_ can strengthen endothelial barrier function via the EP_4_ receptor (Birukova *et al.*, 2007; Konya *et al.*, 2013). It is not clear why there is a difference, but it could be because the cell‐based system does not resemble the complexity of an *in vivo* model or the permeability characteristics of an intact microvascular endothelium.

In recent years, novel EP_2_ and EP_4_ receptor antagonists that are potent and selective for their respective receptor subtype have emerged and can be used to confirm data generated in developmental knockout mouse strains. Previously, AH6809 had been used by most investigators to examine EP_2_ receptor events, but this also antagonizes EP_1_ and DP_1_ receptors (Jones *et al.*, 2009; Woodward *et al.*, 2011). However, af Forselles *et al.* (2011 recently described a novel and selective EP_2_ receptor antagonist, PF‐04418948, which was further characterised by our laboratory (Birrell *et al.*, 2013). In these experiments, PF‐04418948 produced a dose‐dependent inhibition of MVL induced by the EP_2_ agonist, ONO‐AE1‐259. In 2010, Chen *et al.* described a novel EP_4_ receptor antagonist, ER‐819762, that was highly selective for EP_4_ receptors in a number of different *in vitro* assays as well as being effective in both mouse and rat models of arthritis and chronic pain. Similarly, ER‐819762 produced a dose‐dependent inhibition of EP_4_‐induced MVL. However, initial experiments demonstrated that neither antagonist alone was able to inhibit PGE_2_‐mediated airway MVL. Interestingly, when both antagonists were administered simultaneously, at the same doses and time points, a decrease in PGE_2_‐induced MVL was evident, with a significant inhibition seen in the trachea and in the bronchi and IPA. This emphasized the possibility for a dual role for EP_2_ and EP_4_ receptors in mediating PGE_2_‐induced MVL responses. Little research has been published on the role of prostanoid receptors of the EP subtype in airway MVL, and so, the data presented herein demonstrate an EP receptor activity profile, which will aid in further understanding its role in airway inflammation.

There is, however, some information available from other organ systems implicating various EP receptors in MVL by evoking vasodilatation and increasing blood flow, an effect that is particularly evident in the skin. In this regard, both EP_2_ and EP_4_ receptor signalling have been implicated in acute skin inflammation by enhancing blood flow in the micro‐environment (Kabashima *et al.*, 2007). It could also be possible that PGE_2_‐induced MVL may be caused by vasodilatation and an increase in blood flow to the airways. There is considerable evidence indicating that EP_2_ and EP_4_ receptors are pivotal in mediating the vasodepressor actions in mouse and human vascular preparations (Kennedy *et al.*, 1999; Zhang *et al.*, 2000; Imig *et al.*, 2002; Davis *et al.*, 2004). However, in the lung, potent vasodilators such as calcitonin gene‐related peptide do not produce MVL or enhance MVL produced by other agents probably because of the greater blood flow to the lung than to the skin, and so, this is less likely (Rogers *et al.*, 1988).

It has been well‐documented that microvascular leak and oedema are prominent features of allergic airway diseases, such as asthma, with high concentrations of plasma proteins being present in airway secretions from acute asthmatic patients (Meerschaert *et al.*, 1999; Kanazawa *et al.*, 2002, 2000; Khor *et al.*, 2009; Tseliou *et al.*, 2012). Having already demonstrated that PGE_2_ induced airway MVL via the EP_2_ and EP_4_ receptors, its role in a murine respiratory model of airway inflammation was then investigated. An increase in PGE_2_ was measureable in lavage fluid 2 and 6 h but not 24 h after OVA challenge. Others have reported increases in PGE_2_ levels in their asthma models (Herrerias *et al.*, 2009; Swedin *et al.*, 2009). MVL appeared to parallel the changes in PGE_2_ levels in that we could detect an increase at 2 h post‐challenge but not 24 h later. Furthermore, as with the exogenous PGE_2_ experiments, it appears that allergen‐induced MVL is via the activity of PGE_2_ on EP_2_ and EP_4_ receptors.

**Figure 10:**
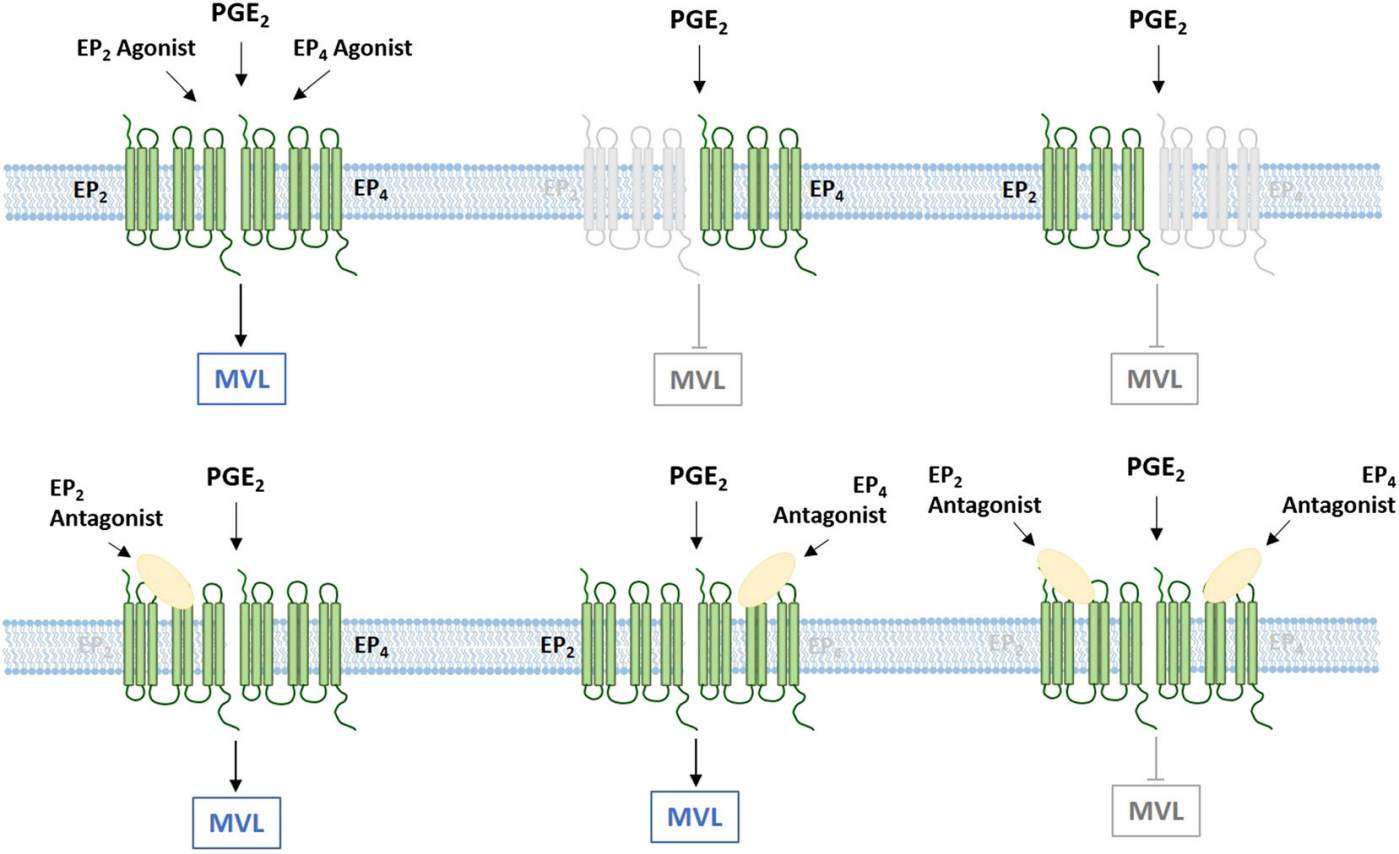
Schematic of possible EP_2/4_ heterodimer and how this interaction influences PGE_2_‐induced airway microvascular leak. (A) PGE_2_ and EP_2_ and EP_4_ agonists are all able to induce a significant increase in airway MVL. However, in EP_2_ and EP_4_ receptor knockouts, this response to PGE_2_ is absent. With either functional receptor missing, the EP_2/4_ heterodimer cannot form, and so, stimulation with PGE_2_ cannot elicit a response. (B) Pharmacological inhibition of either receptor with an antagonist does not inhibit airway MVL. When both EP_2_ and EP_4_ antagonists are administered simultaneously, both receptors are blocked, and PGE_2_‐induced MVL is attenuated.

An interesting observation from the data presented here was that both EP_2_ and EP_4_ agonists caused almost identical increases in airway MVL and, in the knockout mice, responses to PGE_2_ were almost completely abolished in both EP_2_ and EP_4_ receptor knockouts. Moreover, the selective receptor antagonists required simultaneous dosing to block the stimulatory effects of PGE_2_. This suggests that EP_2_ and EP_4_ may be functionally interdependent. A similar effect has been reported in human adipocytes and breast cancer cells with both receptors playing a role in PGE_2_‐induced induction of aromatase and inhibition of either receptor attenuating the effects of PGE_2_ (Subbaramaiah *et al.*, 2008). One hypothesis could be that the EP_2_ and EP_4_ receptors form heterodimers, which has been illustrated in Fig. 10. The data from the knockouts indicate that without functional EP_2_ or EP_4_ receptors, PGE_2_ is unable to induce MVL. Furthermore, when EP_2_ and EP_4_ antagonists were administered alone, when both functional receptors are present, PGE_2_ still induced significant MVL. Only when both receptors were antagonized simultaneously was PGE_2_‐induced leak attenuated.

There is increasing evidence that GPCRs are able to heterodimerize and that this interaction can affect the function of both receptors (McGraw *et al.*, 2006; Wilson *et al.*, 2007). Wilson *et al.* (2007 demonstrated a functionally important heterodimerization of the Tx (TP) and prostacyclin (IP) receptors in human and mouse aortic smooth muscle cells. Additionally, McGraw *et al.* (2006 used fluorescence microscopy in airway smooth muscle cells and BRET and co‐immunoprecipitation in a cell line to show heterodimerization between EP_1_ receptors and β_2_ adrenoceptors. However, more investigations are obviously needed to establish whether this is occurring between the EP_2_ and EP_4_ receptors in this *in vivo* model.

PGE_2_ is a key mediator produced by the lung and has widespread effects according to the EP receptor that is activated. Airway MVL represents a response to injury and under ‘disease’ conditions is a prominent feature of airway inflammation. The data presented here highlight a key role for PGE_2_ and the EP_2_ and EP_4_ receptors. These findings are novel and make an exciting addition to the expanding repertoire of effects mediated by EP receptor activation.

## Author contributions

M. A. B., M. G. B., S. A. M., M. G. and V. O. D. devised the experiments; V. C. J., M. A. B. and M. G. B. wrote the manuscript; V. C. J., S. A. M., S. R. C. and M. G. performed the experiments.

## Conflict of interest

The authors declare no conflicts of interest.
